# Fluorescent Nanocomposite Based on Carbon Quantum Dots for the Quantification of Permanganate Ions in Aquatic Environments

**DOI:** 10.1021/acsomega.6c04511

**Published:** 2026-07-15

**Authors:** Ana Stephani Silva de Lima, Elayne Freitas de Carvalho, Carlos Mateus Paiva Oliveira, João Paulo de Sousa Ferreira, Renato Altobelli Antunes, Thiago Alves de Moura, Alexandre Rocha Paschoal, Juan Simón Rodriguez Hernández, Carlos William de Araújo Paschoal, Alejandro Pedro Ayala, Rafael Melo Freire, Pierre Basílio Almeida Fechine, Samuel Veloso Carneiro

**Affiliations:** † Advanced Materials Chemistry Group (GQMat), Department of Analytical Chemistry and Physical Chemistry, 28121 Federal University of Ceará − UFC, Campus do Pici, CP 12100, Fortaleza, Ceará CEP 60451-970, Brazil; ‡ Theoretical Chemistry Group (GQT), Department of Analytical Chemistry and Physical Chemistry, Federal University of Ceará − UFC, Campus do Pici, CP 12100, Fortaleza, Ceará CEP 60451-970, Brazil; § Center for Engineering, Modeling and Applied Social Sciences, Federal University of ABC, Santo André, São Paulo CEP: 09210-580, Brazil; ∥ Department of Physics, Federal University of Ceará − UFC, Campus do Pici, CP 12100, Fortaleza, Ceará CEP 60451-970, Brazil; ⊥ Laboratory of Nanomaterials Chemistry (LQN), Centro de Investigación En Ingeniería de Materiales (CIIMAT) y CEDENNA, Universidad Central de Chile, Santiago CP 8330601, Chile

## Abstract

This study highlights the development of a sensor based on a PVA/CQDs nanocomposite, which exhibited excellent sensitivity toward the permanganate ion (MnO_4_^–^), making it possible to apply it in aquatic matrices intended for aquaculture activities, ensuring the monitoring of the analyte. For the development of the sensing platform, fluorescent nanoparticles named carbon quantum dots (CQDs) were synthesized and their optical properties were investigated. Real sample assays were performed and the nanoparticles proved to be selective toward the MnO_4_^–^, making it possible to obtain high recovery results. Furthermore, the development of the detection platform becomes an excellent alternative for rapid, simple, and low-cost monitoring for the proper control of MnO_4_^–^ use.

## Graphical abstract

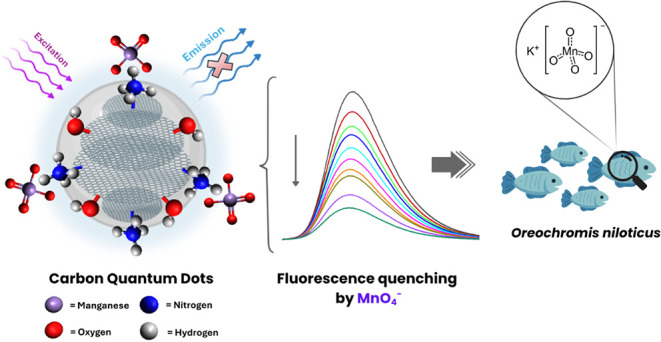

## 1 Introduction

Potassium permanganate (KMnO_4_) is a chemical compound widely used as an oxidizing agent, exhibiting antimicrobial and antiparasitic properties.^1,2^ In aquaculture, it can be applied to improve water quality, disinfect tanks, and control parasites, as it has the potential to oxidize organic matter and reducing compounds such as hydrogen sulfide and ferrous iron, in addition to increasing dissolved oxygen in water through hydrolysis reactions.^3^ Despite its significant sanitary relevance, it is necessary to monitor the use of this chemical species, given that its high oxidative potential can cause changes in the quality of life of the organisms present, thereby affecting ecological processes.

The indiscriminate use of KMnO_4_ at high concentrations can be toxic to species such as tilapia fingerlings *Oreochromis niloticus* at levels equivalent to 1.81 mg L^–1^. Furthermore, KMnO_4_ concentrations above 0.12 mg L^–1^ can cause chronic toxic effects in nontarget organisms, such as the microcrustacean *Ceriodaphnia dubia* and the green microalga *Pseudokirchneriella subcapitata*, threatening the balance of the aquatic food chain.^3^ Currently, several techniques can be employed for the detection of the MnO_4_^–^ ion, such as redox titration, spectrophotometry, and chemiluminescence methods.^4^ However, such methods present some disadvantages, including the requirement for expensive equipment, trained professionals, and the performance of lengthy and complex analyses. Therefore, the development of low-cost methodologies based on nanomaterials with easier and faster detection has become an alternative of great interest.^5,6^

Structures such as nanomaterials have gained space in various processes aimed at sustainability due to some of their properties, such as surface-to-volume ratios, electrical conductivity, and adjustable optical properties, which enable the identification and monitoring of environmental elements such as air and water quality.^7^ Currently, with the advancement of society, one of the main problems to be addressed is environmental pollution. The constant presence of emerging contaminants such as microplastics, potentially toxic compounds, and organic pollutants in aquatic matrices, for example, causes harmful effects on ecosystems, making it necessary to develop simple and selective sensing strategies.^8^

Consequently, Carbon Quantum Dots (CQDs), zero-dimensional nanomaterials, have been gaining prominence in areas such as chemical sensing. They are easily available, can be obtained from natural and synthetic sources, are economical, exhibit low toxicity, and possess good biocompatibility.^9^ One of their main properties is optical, with their principal characteristic being the ability to emit fluorescence.^10^ Due to their optical and electrical properties, CQDs can be used in the fabrication of fluorimetric and electrochemical sensors. Based on the nature and synthesis of these nanoparticles, they can exhibit sensitivity and selectivity toward different contaminant species, such as metal ions,^11^ food additives,^12^ environmental organic pollutants,^13^ and biomolecules,^14^ enabling the detection of such analytes.^15^

The use of these carbon-based nanoparticles has been refined due to the need to replace conventional environmental monitoring methods and as an alternative to the use of inorganic Quantum Dots containing transition metals, which involve high synthesis costs and low biocompatibility. Among the numerous characteristics mentioned previously, such nanostructures can be applied in a wide range of areas, including photocatalysis,^16^ light-emitting diode (LED) fabrication,^17^ bioimaging,^18^ solar cells,^19^ and chemical sensing.^20^

Studies reported in the literature have shown that for manganese species, for example, in high oxidation states, they play an important role in electron transfer and charge separation processes. Thus, it can be observed that in Mn–Co spinel oxides, where redox-active sites favor electron mobility, the permanganate ion can act as an electron acceptor in CQD-based systems, promoting fluorescence quenching.^21^ This feature highlights the relevance of the electronic properties of manganese, both in catalytic applications and in fluorescent sensors.

From this, materials such as polymeric nanocomposites can be synthesized for environmental monitoring applications and are increasingly applied in areas such as sensor manufacturing. Therefore, the incorporation of CQDs to produce thin films, for example, becomes a highly interesting alternative due to the optical properties and biocompatibility of these materials, making them good options for processes that require greater sensitivity and stability, in addition to ensuring better stability in their optical properties. Due to their fluorescent characteristics, CQDs can be incorporated into polymeric materials such as poly­(vinyl alcohol) (PVA) and can be targeted for applications such as the detection of analytes through the fluorescence quenching effects of these nanoparticles, allowing real-time monitoring of potentially toxic compounds and organic pollutants.^22^

Therefore, the present study aims to develop a sensor based on a nanocomposite to ensure the detection and monitoring of the MnO_4_^–^ species in areas intended for aquaculture activities, for which adequate control of the use of such chemical species is necessary. Accordingly, assays with real samples were performed, for which excellent recovery results were obtained, ensuring the reliability of the method. Finally, the developed sensor exhibited sensitivity and selectivity toward the MnO_4_^–^ analyte, showing high potential for application.

## 2 Results and Discussion

### 2.1 CQDs Characterization

Initially, the XPS survey spectra shown in Figure 1a revealed the presence of carbon (72.22%), oxygen (21.09%), and nitrogen (3.02%) atoms in the CQDs nanostructure. From the deconvolution of the high-resolution C 1s spectrum (Figure 1b), four peaks corresponding to carbon bonds were assigned as follows: C–C/C=C (25.24%), C–N/C–O (44.49%), C=O (7.46%), and C=N/O–C=O (3.55%),^24^ with binding energies at 284.15, 285.9, 287.83, and 289.57 eV, respectively. From the deconvolution of the high-resolution O 1s spectrum, functional groups such as C=O (63.47%) and C–O/OH (19.77%) were identified, with binding energies at 534.18 and 532.29 eV, respectively. In addition, a binding energy at 529.72 eV corresponded to adsorbed O_2_ (16.76%),^25^ as shown in Figure 1c. Finally, from the high-resolution N 1s spectrum, N=C (14.55%) and NH_2_/NH_3_^+^ (85.45%) bonds were assigned to the respective binding energies at 399.67 and 402.33 eV, presented in Figure 1d.

**Figure 1:**
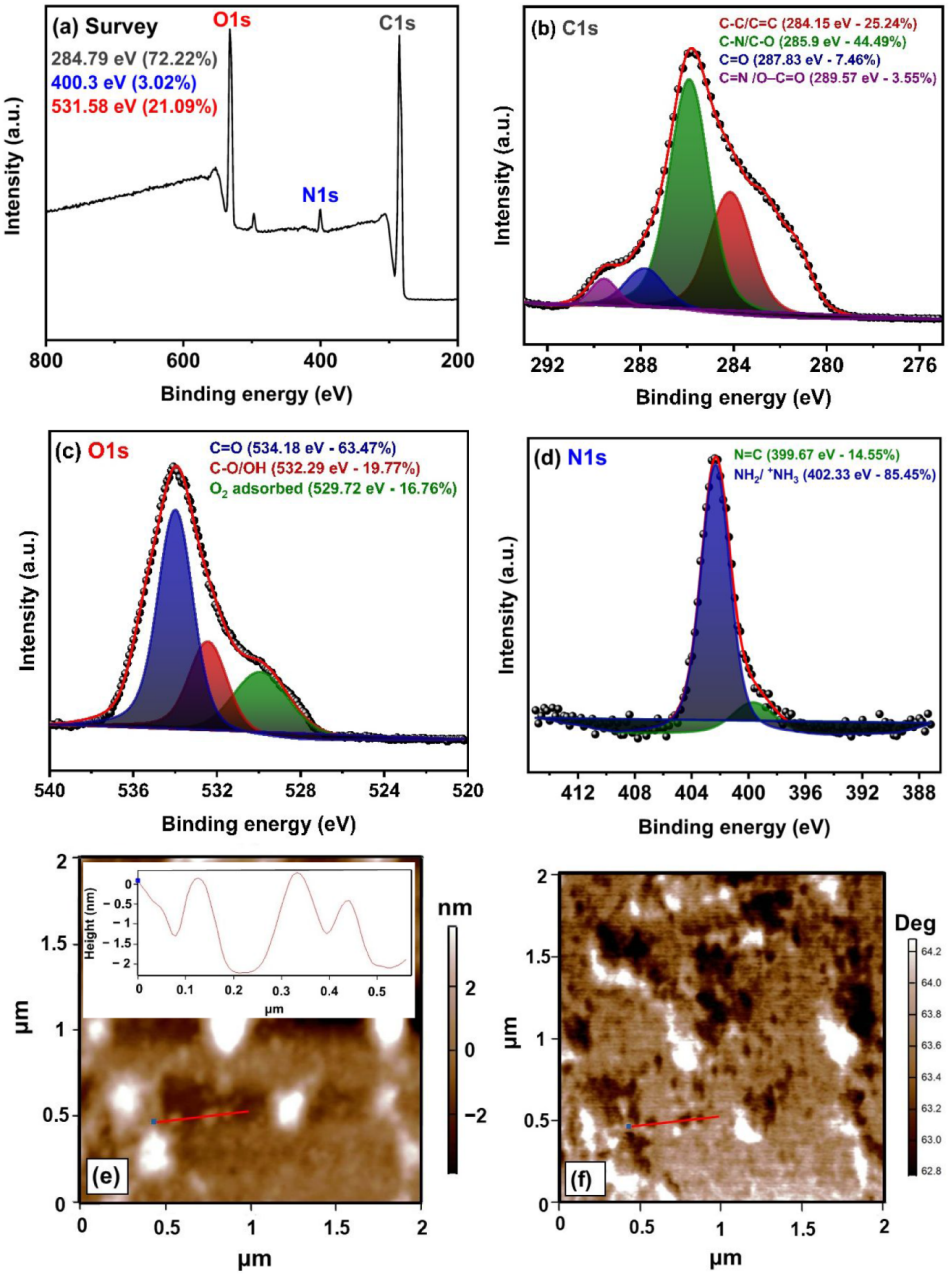
XPS spectra and their deconvolutions of CQDs: (a) Survey; (b) High-resolution spectra of C 1s; (c) O 1s, and (d) N 1s. AFM results: (e) Topographic image and height profile (inset); (f) Phase.

To complement the information on the surface chemistry of the CQDs, FTIR characterization was also employed (Figure S1). The spectrum revealed the presence of bands at 3371 and 3273 cm^–1^, corresponding to O–H and N–H bonds, respectively.^26^ Furthermore, bands at 1660 and 1562 cm^–1^ were attributed to C=O and N–H bonds.^27^ Additionally, a band at 1485 cm^–1^ related to C–N type bonds was also identified.^28^ These assignments obtained by FTIR corroborate the results observed by XPS spectra.

To further investigate the morphology of the CQDs, atomic force microscopy (AFM) was employed. Through this technique, information was also obtained regarding the size of some individual nanoparticles. Based on this analysis, it was possible to observe nanoparticles with nearly spherical morphologies, as shown by the phase image, and diameters of approximately 1.5 nm, observed from the topographic height profile (Figure 1e, inset). These results corroborate other studies found in the literature, in which the synthesized CQDs presented average sizes of 1 to 3 nm.^29^ Additionally, measurements were performed using transmission electron microscopy (TEM). Figure S2a shows an almost spherical morphology of the particles, similar to the images from AFM. However, the size distribution in Figure S2b reveals an average particle size of 0.991 ± 0.261 nm, slightly smaller than that obtained by AFM. Since the size of 100 particles was measured randomly, this result is statistically more reliable, and we can infer that the average size of the CQDs synthesized in this work is approximately 1 nm.

For the optical characterizations of the Carbon Quantum Dots, molecular absorption spectroscopy in the ultraviolet–visible region (UV–vis) was performed (Figure 2a). Two absorption bands were obtained: one located in the 240 nm region, corresponding to π–π* transitions of −C=C bonds, and another at 350 nm, indicating n−π* type transitions of −C=O, −C–N, and C–OH bonds.^30^ Furthermore, the CQDs were also characterized by fluorescence spectroscopy, exhibiting an emission behavior at 450 nm and showing fluorescence in the blue region of the visible spectrum. When subjected to excitation at different wavelengths (300–400 nm), the CQDs demonstrated an excitation wavelength-independent emission behavior (Figure S3), possibly indicating a nanoparticle with an ordered and homogeneous graphitic structure.^31^ These characteristics depend on the adjustments made to the synthesis conditions, such as temperature and pressure, which can lead to changes in the nanoparticle structure, size, and surface composition, as well as in their physical and chemical properties.^32^

**Figure 2:**
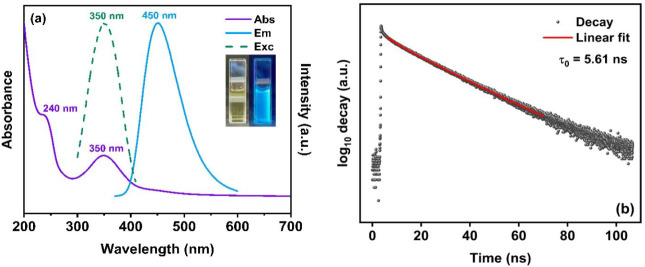
Spectroscopic characterizations: (a) UV–vis spectra, emission, and excitation; (b) Fluorescence lifetime of CQDs.

In addition, the average fluorescence lifetime of the nanoparticles was also measured (Figure 2b). By analyzing the lifetime of a fluorophore, it is possible to determine the time required for electrons to return from the excited state to the ground state, emitting energy in the form of photons. The result obtained for the average lifetime of the CQDs synthesized was 5.61 ns. This analysis was performed based on radiative decay using a biexponential function. This result corroborates several findings previously reported in the literature, in which fluorophores with considered long lifetimes (4 – 5 ns) can be associated with the surface defects present in the synthesized CQDs.^33^

The quantum yield (QY) is another parameter that is directly related to the presence of heteroatoms on the surfaces of the CQDs. Elements such as nitrogen and phosphorus, for example, can readily bond to the surfaces, improving the QY value and decreasing aggregation among the nanoparticles.^34^ In this work, the quantum yield (QY) value obtained for carbon quantum dots (CQDs) was 19.3% at pH 7.52. This fact can be justified by the presence of ethylenediamine as a precursor during the synthesis of CQDs, suggesting that functionalization with heteroatoms, such as nitrogen, has the potential to alter the surface states of the nanostructures, thereby improving their quantum yields.^35^

### 2.2 Sensing Tests

The first step for optimizing the experimental conditions of the CQDs was the determination of the ideal concentration of nanoparticles. In the spectrofluorimeter, the CQDs sample was subjected to measurements at different concentrations, maintaining an excitation wavelength of 350 nm with a scan from 370 to 600 nm. Based on this analysis, it was observed that the fluorescence intensity of the CQDs decreased with the reduction in concentration, while the emission maximum remained at 450 nm (Figure 3a). During the experiment, it was observed that increasing the concentration of CQDs led to results that did not exhibit linear behavior (Figure S4), indicating possible aggregation among the nanoparticles themselves. Therefore, 0.09 mg mL^–1^ was established as the ideal concentration, which corresponds to the midpoint located on the line of the linearized graph (Figure 3b). This value was determined with the aim of using small amounts of material while still maintaining a high fluorescence intensity.

**Figure 3:**
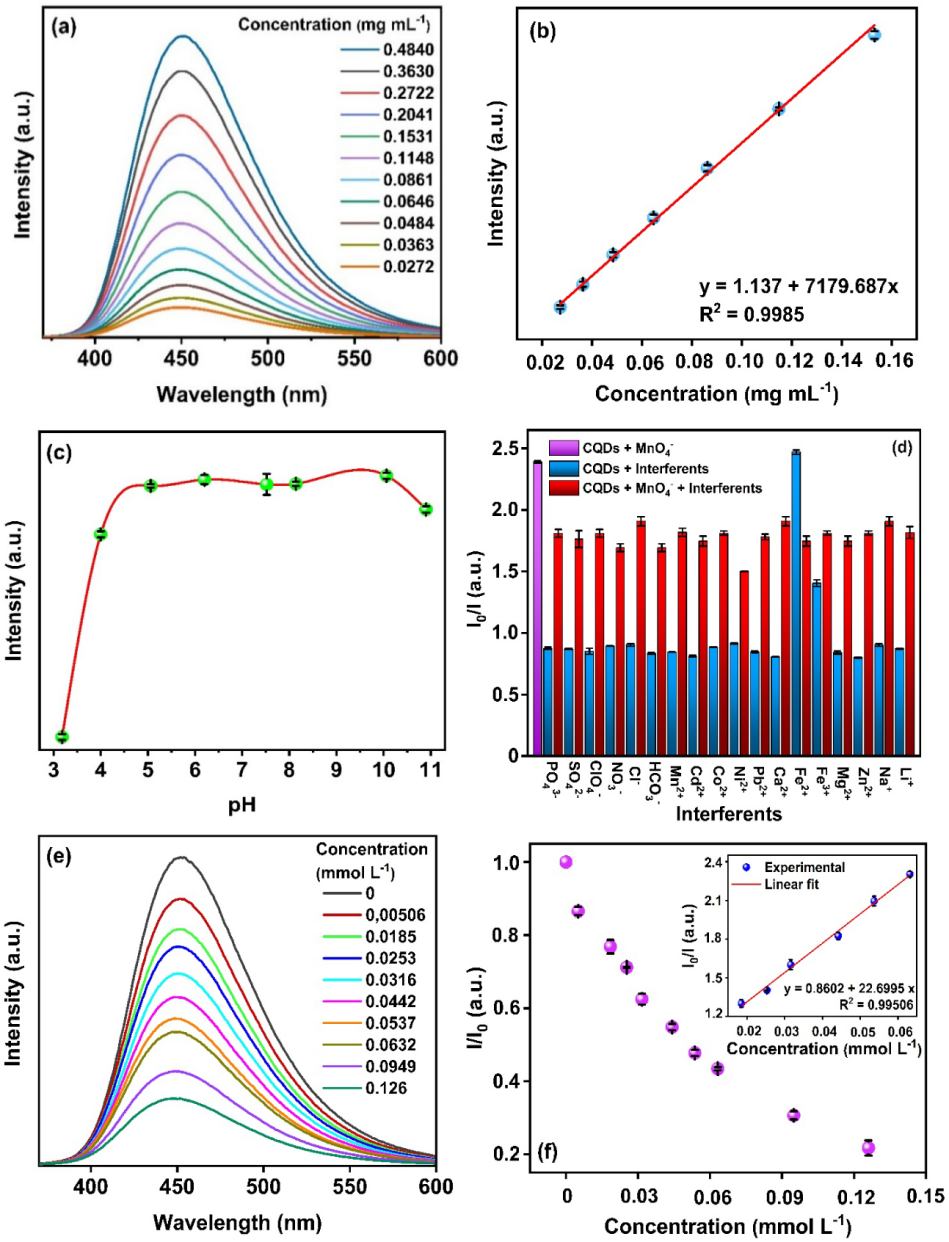
(a) Fluorescence emission spectrum of CQDs at different concentrations; (b) Graph of fluorescence emission as a function of concentration at 450 nm; (c) Effect of different pH on fluorescence emission intensity; (d) Effects of interferents on fluorescence intensity in the presence and absence of MnO_4_^–^ ion; (e) Calibration curve at different concentrations of MnO_4_^–^; (f) Linearized graph of fluorescence quenching as a function of different MnO_4_^–^ concentrations.

For the pH tests, the CQDs were subjected to pH values ranging from 3 to 11 (Figure 3c), revealing a decrease in fluorescence intensity in highly acidic media, with possible aggregation among the nanoparticles. The opposite effect can be observed in more alkaline environments, where the deprotonation of functional groups (carboxylates, for example) occurs on the CQDs’ surface. In the present study, in the pH range of 6–8, the nanoparticles exhibited the best fluorescence emission stability. Indeed, the presence of functional groups such as carboxylic acids, hydroxyls, amines, among others, can lead to variations in the fluorescence emission intensity of CQDs when subjected to different pH values.^36^ In highly acidic environments, the nanoparticles may aggregate, causing self-quenching of their fluorescence.

The selectivity of the CQDs toward the MnO_4_^–^ ion was evaluated using a standardized KMnO_4_ solution with a concentration of 78.9 mmol L^–1^. To confirm this selectivity specifically toward the MnO_4_^–^ ion, the fluorescence emission behavior was evaluated only in the presence of the Mn^2+^ species, which showed no interference results or selectivity by the CQDs. The fluorescence intensity of the CQDs was also observed in the presence of some possible species present in the aquatic matrix under analysis: Na^+^, Li^+^, Pb^2+^, Ni^2+^, Co^2+^, Cd^2+^, Cl^–^, NO_3_^–^, SO_4_^2–^, PO_4_^3–^, ClO_4_^–^, HCO_3_^–^, Ca^2+^, Fe^2+^, Fe^3+^, Mg^2+^, Zn^2+^. Therefore, an initial study was conducted to evaluate the fluorescence quenching of the nanoparticles in the presence of such interfering species and to analyze their fluorescence quenching behavior toward the MnO_4_^–^ ion. Subsequently, a similar assay was performed, aiming to prove the selectivity of the nanoparticles exclusively for the MnO_4_^–^ analyte when in contact with those previously mentioned interfering ions (Figure 3d). For the interference assays, in the absence of the MnO_4_^–^ ion, fluorescence quenching of the nanoparticles by Fe^2+^ and Fe^3+^ ions was observed. This phenomenon may occur due to electrostatic interactions between the ions and the nanoparticles, leading to their aggregation, as well as possible redox interactions between the CQDs and iron.^37^ Given this result, it is important to emphasize that if any humic sample is analyzed, pretreatment of the sample is necessary to separate the Fe^2+^ and Fe^3+^ ions so that these species do not interfere with the result of the analysis, which would lead to a false positive.

In this experiment, solutions of interfering ions were used at a known concentration of 0.2 mg mL^–1^. It is noteworthy that at this concentration, the highest fluorescence quenching by the MnO_4_^–^ ion was observed.

After characterizing the optical properties of CQDs and optimizing the experimental conditions of the system, sensing tests were performed. Initially, a calibration curve was constructed using the CQDs at their ideal concentration of 0.09 mg mL^–1^, an initial KMnO_4_ solution with a concentration of 0.6326 mmol L^–1^, in phosphate buffer solution at pH 7.4. The fluorescence behavior of CQDs in the presence of MnO_4_^–^ was observed in the concentration range of 0.00506 mmol L^–1^ to 0.126 mmol L^–1^. Therefore, the synthesized nanoparticles, in addition to exhibiting high selectivity for MnO_4_^–^ ion, had their fluorescence quenched to the maximum at a concentration as low as 0.126 mmol L^–1^, as shown in Figure 3e.

From this experiment, it was also possible to determine the limit of detection (LOD), which corresponds to the lowest detectable concentration, and the limit of quantification (LOQ), referring to the lowest quantifiable concentration of the analyte.^38^ These can be obtained through the following eqs 1 and 2, respectively:

1 $$LOD=3,3\frac{\sigma }{S}$$

2 $$LOQ=10\frac{\sigma }{S}$$

where σ corresponds to the standard deviation of the response and *S* to the slope of the analytical curve obtained from the experiment. For the determination of the LOD and LOQ results, a linear relationship (*I*_0_ */I*) was established based on the concentration range of MnO_4_^–^ for which the smallest deviation from linearity was detected (Figure 3f).

Thus, values of 1.009 mg L^–1^ were obtained for the limit of detection and 3.058 mg L^–1^ for the limit of quantification of the analyte under analysis. These results, when compared with literature, fall within an acceptable range for the detection of the MnO_4_^–^ ion, since the development of fluorescent sensors for the target analyte is the subject of increasing study.^39^ Based on this, the results obtained for the present study can be compared with other works reported in the literature focusing on the development of fluorescent sensors using CQDs (Table 1), applied to water quality monitoring and soil analysis,^40–42^ metal ion detection, and dye stabilization^43^ through analyses employing the MnO_4_^–^ ion.

**Table 1:** Comparative Studies of Fluorescent Probes Using CQDs for the Detection of MnO_4_^–^ Ion

Reference	Method	LOD	Linear Range	Application
This work	Solvothermal	6.39 μmol L^–1^	18.5 – 94.9 μmol L^–1^	Fish tanks
^40^	Hydrothermal	0.0418 μmol L^–1^	0.5 – 168 μmol L^–1^	Water
^41^	Hydrothermal	0.640 μmol L^–1^	1 – 60 μmol L^–1^	Tap water/Lake water
^42^	Hydrothermal	0.06 μmol L^–1^	0 – 50 μmol L^–1^	Water/Soil
^43^	Hydrothermal	1.2 μmol L^–1^	0 – 20 μmol L^–1^	Detection of metal ions/Stabilization of dyes

### 2.3 Fluorescence Quenching Mechanisms and Thermodynamic Analysis

The thermodynamics of the interaction between CQDs and MnO_4_^–^ ions were carefully studied. In this way, calibration curves with the MnO_4_^–^ ion were performed at three different temperatures: 298.15, 300.15, and 303.15 K. The values of the parameters obtained from the Stern–Volmer equation of each experiment are shown in Table 2. Concerning parameter *n*, when *n* values equal or close to 1 are obtained, it can be concluded that the interactions between the fluorophore and the quencher occur at only one interaction site.^44^

**Table 2:** Parameters Obtained from the Stern–Volmer Equation for Three Different Temperatures

Temperature (K)	(K_SV)_	*n*	R^2^
298.15	48.20065 ± 0.51	1.81426 ± 0.04	0.99765
300.15	30.23527 ± 0.87	1.37238 ± 0.10	0.97798
303.15	23.7619 ± 0.80	1.27871 ± 0.12	0.96893

From the results obtained for the *n* values in the present experiment, which were 1.81426 ± 0.04, 1.37238 ± 0.10, and 1.27871 ± 0.12 at different temperatures of 298.15, 300.15, and 303.15 K, respectively, it is possible to observe that *n* is greater than 1, indicating the occurrence of both static and dynamic quenching mechanisms and suggesting the presence of multiple interaction sites between the nanoparticles and the MnO_4_^–^ ion.^45^ Furthermore, the predominance of the static mechanism is evident, since K_SV_ decreases as the system temperature rises (Figure 4a, inset), indicating the formation of a nonfluorescent complex between the nanoparticles and the quencher.^45^

**Figure 4:**
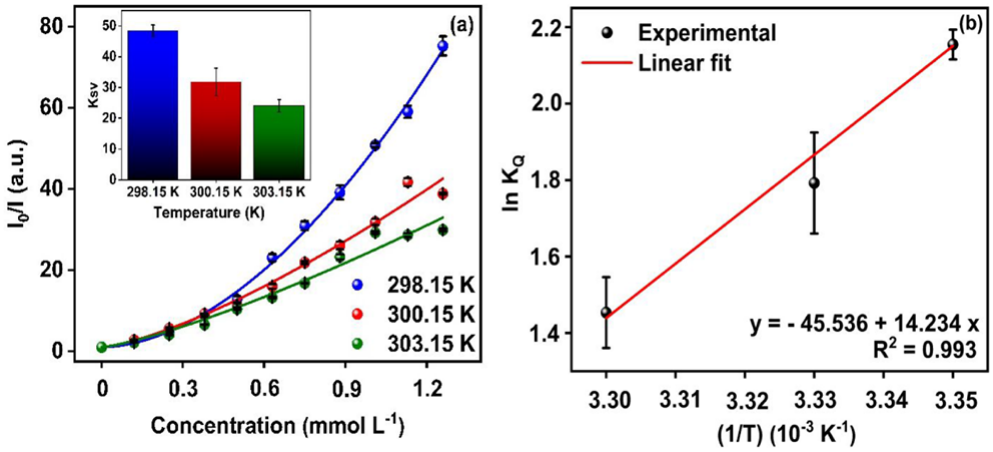
(a) Effect of temperature on the fluorescence intensity of CQDs, based on the Stern–Volmer equation; (b) Graph obtained from the van’t Hoff equation.

Through the van’t Hoff equation for the description of the equilibrium of the CQDs + MnO_4_^–^ system, the thermodynamic state functions were determined: standard enthalpy change (ΔH°), standard entropy change (ΔS°), and standard Gibbs energy change (ΔG°), as presented in Figure 4b and Table 3.

**Table 3:** Thermodynamic Parameters Obtained Using the van’t Hoff Equation

	Temperature (K)		
Parameters	298.15	300.15	303.15
ΔH° (J mol^–1^)		– 378.58	
ΔS° (J K^–1^ mol^–1^)		118.34	
ΔG° (J mol^–1^)	– 5340.58 ± 1.08	– 4472.63 ± 0.01	– 3663.07 ± 1.1
*K*_*Q*_	8.628 ± 0.33	6.038 ± 0.80	4.292 ± 0.40
*n*	1.81426 ± 0.04	1.37238 ± 0.10	1.27871 ± 0.12

Therefore, it is possible to observe the decrease in the *K*_*Q*_ values with increasing temperature;^46^ furthermore, the positive linear trend of *ln K*_*Q*_ versus *1/T* confirms the formation of a stable nonfluorescent complex. In addition to the presence of static and dynamic mechanisms, the fluorescence enhancement or quenching of CQDs can also be related to the presence of one or more quenching mechanisms, such as the inner filter effect (IFE), fluorescence resonance energy transfer (FRET), photoinduced electron transfer (PET), among others. IFE occurs when the absorption spectra of the quencher precisely overlap with the excitation or emission spectra of fluorophore materials (Figure S5).^47^

Based on the functional groups identified through structural characterizations and the identification of the predominance of static and dynamic quenching mechanisms, in addition to the presence of IFE, we proposed a hypothesis for the fluorescence quenching mechanism of CQDs in the presence of the MnO_4_^–^ ion presented in Scheme 1. Through XPS and FTIR spectra, the presence of amino-type groups was verified, which was expected due to functionalization with ethylenediamine during the nanoparticle synthesis. Furthermore, the sensitivity of CQDs to pH changes demonstrates that protonated or deprotonated species may exist in their composition.

**Scheme 1:**
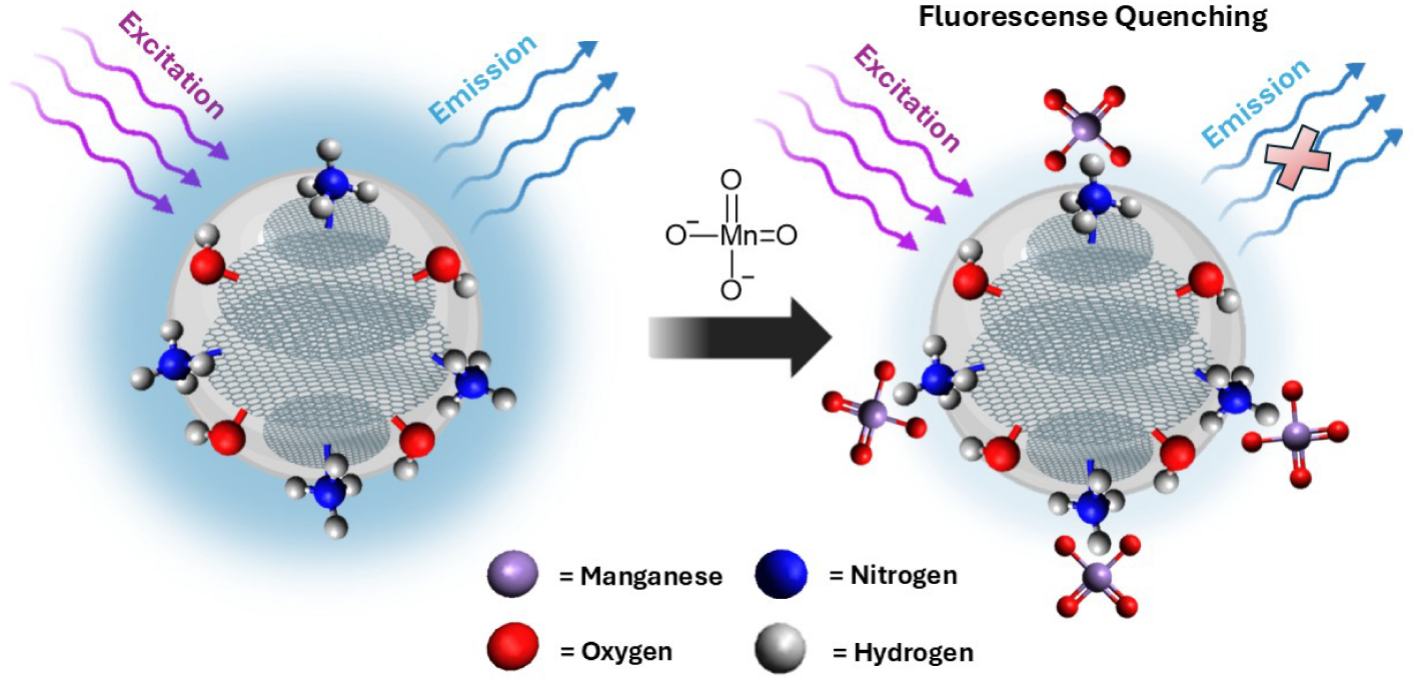
Schematic Representation of the Fluorescence Quenching Mechanism of CQDs in the Presence of MnO_4_^–^ ^a^

a The characteristic emission of CQDs upon excitation is diminished by interaction with MnO_4_^–^ ions, indicating a quenching mechanism governed by electrostatic interactions and potentially involving the formation of a non-fluorescent complex.

Indeed, since the analyses were performed at pH 5.8 and under phosphate buffer, a protonated surface with NH_4_^+^ groups is expected in the nanoparticles. Therefore, it is possible to propose the hypothesis that an electrostatic attraction occurs between the amino groups upon contact with the MnO_4_^–^ ion, forming a nonfluorescent type of complex between the nanoparticles and the quencher.^41^ Furthermore, due to the oxidizing potential of the MnO_4_^–^ ion, it can interact with other functional groups, such as hydroxyls, contributing to fluorescence quenching.

### 2.4 Tests with Real Samples

For the recovery assays, a water sample was collected from a water reservoir intended for the farming of fish species such as tilapia, located in the city of General Sampaio, Ceará. The real sample was diluted 500-fold with ultrapure water and then doped with different concentrations of MnO_4_^–^: 0.00674 mmol L^–1^; 0.0253 mmol L^–1^; and 0.126 mmol L^–1^. A 500-fold dilution was performed, primarily to mitigate the effects of potential interfering species. The complexity of environmental matrices can significantly affect the detection and behavior of chemical species,^48^ as interfering species can influence the fluorescence signal through various mechanisms, including nonspecific quenching and the inner-filter effect. This dilution step considerably attenuates such interferences, thereby enhancing the method’s selectivity. Furthermore, it ensures that the MnO4^–^ concentration falls within the analytical method’s linear dynamic range, guaranteeing the reliability of the measurement.

The recovery results were obtained using the spectrofluorimeter as the analysis instrument, with six replicates performed for each chosen analyte concentration. These results are shown in Table 4.

**Table 4:** Recovery Results (%) Obtained from Three MnO_4_^–^ Concentrations Selected through the Calibration Curve

Added (mmol L^–1^)	Found (mmol L^–1^)	RSD (Relative Standard Deviation)	Recovery (%)	RSD (Relative Standard Deviation)
0.00674	0.007098	± 3.82E^–5^	105.28	± 0.567
0.0253	0.024217	± 0.00011	95.72	± 0.446
0.126	0.122384	± 0.00096	97.13	± 0.766

Based on the recovery results obtained, which were 105.28, 95.72, and 97.13%, respectively, it can be inferred that the analytical method employed exhibits good reliability for the detection of MnO_4_^–^ in the analyzed aquatic matrix and in other similar matrices, as they fall within the 95% to 105% range, which is considered an acceptable result in the literature.^49^ Furthermore, the aim is to obtain a recovery close to 100%, as this makes it possible to prove that the analytical method used is suitable and capable of detecting the presence of the analyte in question. Additionally, the deviations obtained were considered low and acceptable, which reinforces the reliability and reproducibility of the data.

### 2.5 PVA/CQD Sensor

After evaluating the sensing performance of MnO_4_^–^ ions in aqueous suspension, a sensor based on a polymeric film of PVA and CQDs was produced. The incorporation of the nanoparticles into the polymer was proposed with the aim of ensuring their stability in the solid state rather than in solution, making it viable for storage and distribution.^50^ The proposed approach solves the main challenge of many field sensors, such as the instability of liquid reagents or the complexity of immersion sensors. For the development of the sensor, PVA was the polymer selected due to its solubility characteristics, making it an excellent alternative for the long-term storage of the nanoparticles. To obtain the fluorescence quenching analysis using the *turn-off* sensing method, a spectrofluorimeter was employed to perform the measurements.

Briefly, a procedure similar to the one previously performed was adopted for sensing with the PVA/CQDs nanocomposite. Thus, the fluorescence spectrum of PVA/CQDs was recorded in the absence of the analyte. Then, known concentrations of a KMnO_4_ solution were added. From the results obtained in Figure 5a, it was possible to verify the feasibility of using the sensor, as upon the addition of increasing analyte concentrations, the fluorescence of the nanoparticles was reduced, demonstrating the *turn-off* sensing behavior.

**Figure 5:**
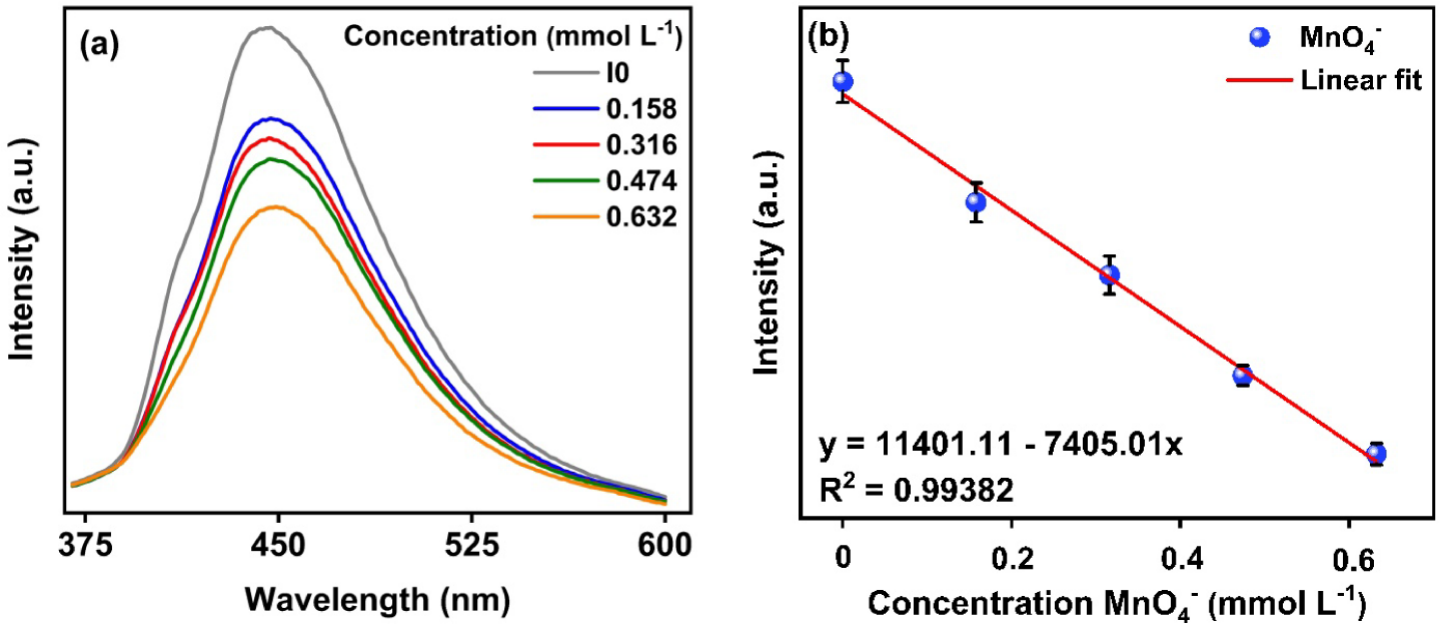
(a) Fluorescence emission spectra of the PVA/CQDs-based sensor recorded at the maximum emission wavelength (450 nm) under 350 nm excitation, at different MnO_4_^–^ concentrations; (b) linearized calibration plot showing the fluorescence emission of the PVA/CQDs sensor as a function of decreasing MnO_4_^–^ concentration.

This behavior can be associated with findings from several studies reported in the literature, which show that in composites such as carbon black and nitrile rubber, where mechanical properties depend on interactions between the carbon phase and the polymer matrix, the performance of Carbon Quantum Dots as fluorescent sensors can be observed through interactions between their surface groups and the chemical species present in the medium.^51^

Furthermore, the linear behavior illustrated in Figure 5b showed satisfactory results with an R^2^ value of 0.99382, indicating that the developed detection platform is in agreement with the Stern–Volmer model, which allows for the elucidation of the fluorescence quenching mechanism between the nanoparticles and the quencher. Additionally, the obtained linearity demonstrates the presence of a constant quenching effect across the entire analyte concentration range used.

Moreover, the LOD and LOQ values for the PVA/CQDs platform were calculated to be 52.518 mg L^–1^ and 159.147 mg L^–1^, respectively. From these results, it can be inferred that the obtained LOD value is higher than expected for determining concentration levels considered toxic to the species *Oreochromis niloticus*. However, the developed sensing platform can still be applied in areas intended for the cultivation of these species, such as in rearing tanks where high concentrations of KMnO_4_ may be used, allowing for evaluation through process monitoring due to the potential accumulation of the analyte. Despite the reduction in sensor sensitivity, it still ensures reproducibility and stability from the nanoparticles, and it still exhibited a fluorescence response to the MnO_4_^–^ ion, displaying a *turn-off* sensing behavior. Furthermore, it is important to highlight that the nanocomposite was designed with the primary goal of ensuring the structural stability of the nanoparticles; however, the use of the aqueous CQDs probe in the absence of PVA is also not ruled out.

In fish farming activities, the sensor can be reproduced using water samples from Tilapia breeding sites, reinforcing the efficiency demonstrated by the previously performed procedure. Reproducibility is achievable through portable fluorometer instruments suitable for field use, enabling rapid measurements and ease of operation, thereby eliminating the requirement for laboratory analysis with expensive equipment like spectrofluorimeters and specialized personnel.

## 3 Conclusion

In summary, this paper suggests a sensor based on a nanocomposite of CQDs and PVA for the detection of the MnO_4_^–^ ion in aquatic matrices. These environments are common in fish farming, such as tilapia. The strategy for sensing and detecting the contaminant was carried out using the *turn-off* mechanism, based on the fluorescence quenching of the synthesized nanoparticles, which undergo simultaneous static and dynamic quenching effects, in addition to the presence of the inner filter effect. Beyond obtaining the limits of detection and quantification, which were 1.009 mg L^–1^ and 3.058 mg L^–1^, respectively, assays with the real sample were performed, making it possible to obtain satisfactory recovery results, ensuring the reliability of the employed method. Consequently, the monitoring of MnO_4_^–^ can be carried out quickly and without the need for long and complex analyses, providing better control over its use and ensuring the quality of life of the aquatic species present.

## 4 Materials and Methods

### 4.1 Materials and Reagents

Citric acid (C_6_H_8_O_7_ 99.5%, Vetec), ethylenediamine (C_2_H_8_N_2_, 98.0%, Vetec), absolute ethanol (CH_3_CH_2_OH, 99.8%, NEON), sodium hydroxide (NaOH, 98.0%, Synth), hydrochloric acid (HCl, 36.5–38.0%, Dinâmica), distilled water (resistivity 333.3 Ω m at 22.1 °C), potassium permanganate (KMnO_4_ P.A., 100.0%, Synth), sodium oxalate (Na_2_C_2_O_4_, 99.5%, Vetec), sulfuric acid (H_2_SO_4_, 100%, Synth), sodium chloride (NaCl, 99.0%, Merck), copper­(II) sulfate (CuSO_4_, 99%, Dinâmica), cadmium chloride monohydrate (CdCl_2_·H_2_O, 99%, Vetec), nickel­(II) chloride hexahydrate (NiCl_2_·6H_2_O, 99%, Dinâmica), lead­(II) nitrate (Pb­(NO_3_)_2_, 99%, Vetec), lithium chloride (LiCl, 99.5%, Synth), cobalt­(II) chloride hexahydrate (CoCl_2_·6H_2_O, 98.0%, Dinâmica), sodium nitrate (NaNO_3_, 99%, Dinâmica), sodium sulfate (Na_2_SO_4_, 99%, Synth), manganese­(II) chloride tetrahydrate (MnCl_2_·4H_2_O, 99%, Synth). For the recovery tests, the water sample was collected from the reservoir in the city of General Sampaio with geographical coordinates 4°4′50.88″S, 39°27′27.65”W, in Ceará, and ultrapure water was obtained from a Milli-Q system (resistivity 1250 Ω m at 22.0 °C). For the purification of the CQDs, dialysis was performed using a Spectra/Por 6 membrane with a molecular weight cut-off (MWCO) of 1 kDa. The experiments were conducted in phosphate buffer solution prepared from sodium phosphate monobasic (NaH_2_PO_4_, 99.0%, Vetec) and sodium phosphate dibasic dodecahydrate (Na_2_HPO_4_·12H_2_O, 99.0%, Vetec).

### 4.2 CQD Synthesis

For the synthesis of CQDs, a solvothermal method was chosen, using citric acid and ethylenediamine as precursors. From this, 1.0 g of citric acid (5.2 mmol) was weighed and dissolved in 9 mL of ethanol. The solution was solubilized for 5 min under magnetic stirring at room temperature. Next, 1 mL of ethylenediamine (15 mmol) was added to the previous solution, instantly resulting in a cloudy appearance. After this procedure, another aliquot of 10 mL of ethanol was added. The solution was stirred for 30 min at room temperature and then placed in a Teflon hydrothermal reactor at 200 °C for 3 h. After the synthesis, a dark brown colloidal suspension was obtained, which was cooled to room temperature of 25 °C and stored under refrigeration. The obtained CQDs exhibited a blue coloration when excited by ultraviolet (UV) light and were finally purified by dialysis, which occurred in two cycles, 12 h each (Scheme 2).

**Scheme 2:**
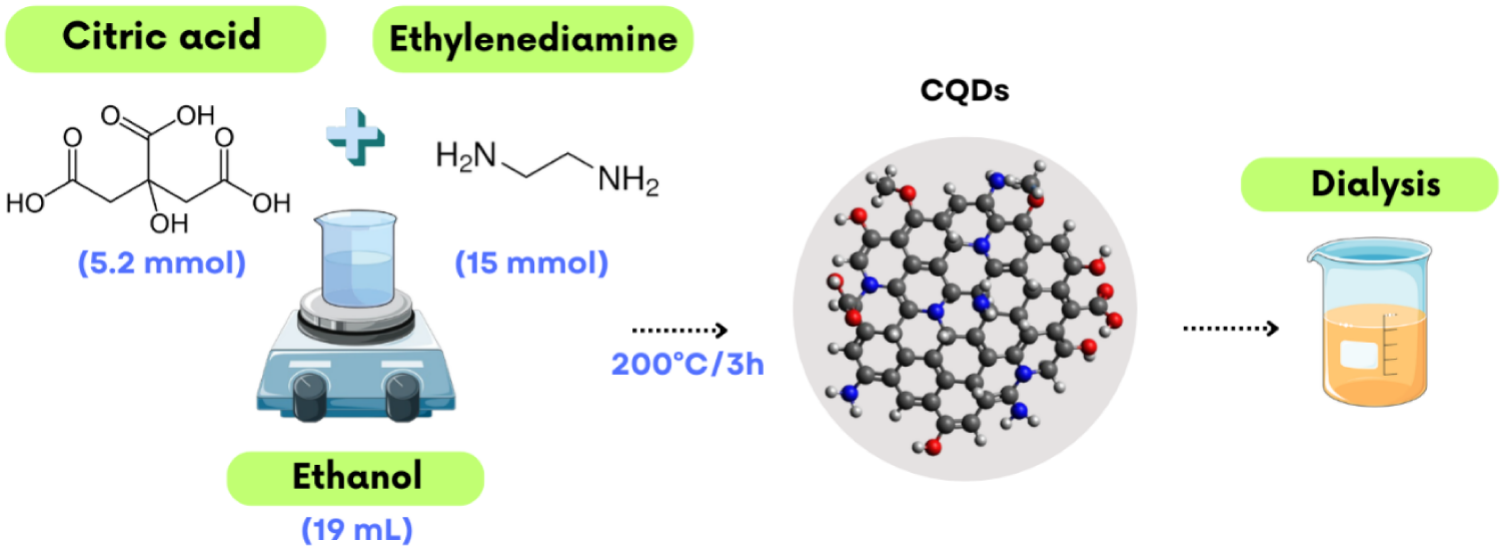
Schematic Representation of the Preparation of CQDs from Citric Acid and Ethylenediamine in Ethanol, Subjected to Heating at 200 °C for 3 h, Followed by Purification via Dialysis[Fn sch2-fn2]

a Icons adapted from Servier Medical Art, licensed under CC BY 3.0.

### 4.3 Instrumentation and Characterization

For the optical characterization techniques of the CQDs, ultraviolet–visible (UV–vis) absorption spectroscopy was performed using a Shimadzu UV-2600 spectrophotometer (wavelength range of 200 to 700 nm). Fluorescence spectroscopy was performed using a Shimadzu RF-6000 spectrofluorophotometer, with excitation readings from 370 to 600 nm. Both characterizations were performed in a quartz cuvette with a 1 cm optical path. For fluorescence lifetime measurements, analyses were conducted in an aqueous medium using a Horiba PPD-900 system coupled to a FiPho detector and a DeltaDiode excitation laser with a wavelength of 405 nm (DD-405L). To obtain the XPS spectra, a ThermoFisher Scientific X-ray photoelectron spectrometer was used, employing monochromatic excitation of Al Kα with a spot size of 10 mm and a passage energy of 46.95 eV. For FTIR analysis of the CQDs and precursors, a Shimadzu IRTracer-100 vibrational spectrometer was used in the 4000 to 400 cm^–1^ range, and the sample was analyzed in KBr pellet form. For microscopic analysis, an Asylum Research MFP-3D atomic force microscope (AFM) was used, which made it possible to obtain the morphology of the nanoparticles and infer their dimensions. The TEM grids were prepared by a drop-casting method. Briefly, a 3 μL aliquot of the properly diluted dispersion was deposited onto a Ted Pella grid (Model 01894, Formvar/carbon-coated 400 mesh copper grid) and allowed to dry completely at room temperature. Transmission Electron Microscopy (TEM) analyses were performed on a Thermo Fisher F200 microscope operated at an accelerating voltage of 200 kV. Images were acquired in parallel-beam bright-field (BF) mode utilizing a CETA-D CMOS camera. A random count of 100 nanoparticles was performed using the ImageJ application.

### 4.4 Fluorescent Quantum Yield

To obtain the quantum yield (QY) of the nanoparticles, eq 3 was used:

3 $$QY=QY_{ref}\left(\frac{I}{I_{ref}}\right)\left(\frac{A_{ref}}{A}\right){\left(\frac{n}{n_{ref}}\right)}^{2}$$

where *I* corresponds to the integrated area of the emission spectrum, *A* is the absorbance at the excitation wavelength, and *n* is the refractive index of the solvent (distilled water). For the calculation of this property, species with known *QY* values, such as quinine sulfate in sulfuric acid 0.1 mol L^–1^, are used as the reference standard (*ref*), with QY_*ref*_ = 54%.

### 4.5 Sensing Strategy

To optimize the experimental conditions, it was initially necessary to determine the ideal concentration of CQDs. For this, the nanoparticles were subjected to a lyophilization process. The suspension of nanoparticles was frozen in liquid nitrogen; after complete solidification, the sample was placed in a freeze dryer, where it remained for 24 h under a pressure of 100 μmHg and a temperature of −50 °C. Subsequently, we resuspended the nanoparticles in 2 mL of distilled water, and a calibration curve was constructed using UV–vis spectroscopy from absorbance measurements at a wavelength of 350 nm. Then, the nanoparticles were taken to a spectrofluorometer, and a procedure similar to the UV–vis analysis was performed with excitation at 350 nm and emission scanning in the range of 370 to 600 nm.

To determine the influence of pH on the fluorescence intensity of the CQDs, suspensions with pH values from 3 to 11 were prepared using the nanoparticles at their ideal concentration. For the preparation, aqueous solutions of HCl and NaOH (0.1 mol L^–1^ and 0.01 mol L^–1^) were required to perform the adjustments.

Initially, a potassium permanganate (KMnO_4_) solution was standardized to obtain the analytical curve. In this sense, a redox titration with sodium oxalate (Na_2_C_2_O_4_) and sulfuric acid (H_2_SO_4_) was performed to remove manganese dioxide (MnO_2_). Briefly, 0.50 g (3.73 mmol) of sodium oxalate (previously dried in an oven at 105 °C) was weighed. Subsequently, 0.15 g (1.11 mmol) of this compound was removed and dissolved in 60 mL of distilled water. The solution was stirred at room temperature until complete dissolution. Afterward, 0.50 g (3.16 mmol) of 20 mmol L^–1^ KMnO_4_ were weighed and dissolved in 150 mL of distilled water. This solution was stirred at a temperature of 60 °C for 1 h. After complete dissolution, the solution was stored in a dark flask to protect it from reducing vapors and light for 3 days at room temperature. Finally, the solution was filtered using a sintered glass funnel to remove all MnO_2_ from the medium. Subsequently, 15 mL of sulfuric acid (H_2_SO_4_) in a 1:8 ratio was heated to 80–90 °C, and the standardization was performed.

Information regarding the selectivity of the fluorescent performance of CQDs was obtained with solutions containing various cations and anions as interferents. Finally, to evaluate the proposed detection of the MnO_4_^–^ ion, a calibration curve was performed at room temperature (25 °C) with MnO_4_^–^ concentrations ranging from 0.00506 mmol L^–1^ to 0.126 mmol L^–1^. Both experiments were conducted keeping the volume of CQDs constant at 500 μL, at its ideal concentration, and in a phosphate buffer solution. For the fluorescence analysis experiment with the real sample, calibration curves were generated with MnO_4_^–^ concentrations of 0.00674 mmol L^–1^, 0.0253 mmol L^–1^, and 0.126 mmol L^–1^. The procedure was performed similarly to previous ones, in which the detection response was obtained by quenching the fluorescence of the nanoparticles.

### 4.6 Development of the PVA/CQDs Sensor

To develop a carbon nanoparticle-based sensor, a polymeric film was produced using poly­(vinyl alcohol) (PVA), a water-soluble synthetic polymer. Approximately 0.5 g of PVA was weighed into a 50 mL beaker. The PVA was then dissolved in 10 mL of a CQDs solution over a period of 3 h, under stirring and at a temperature of 30 °C. Finally, the PVA/CQDs solution was placed in a glass Petri dish and left at room temperature for 48 h to allow drying.

The platform based on PVA/CQDs film was tested using a calibration curve with the MnO_4_^–^ ion, similarly, to obtained in aqueous suspension. For this, the film was dissolved in 20 mL of a phosphate buffer solution at pH 7.4 to ensure optical homogeneity, and the sample was taken to the spectrofluorometer with an excitation wavelength of 350 nm and a scanning range of 370 to 600 nm. The analyses were performed maintaining a fixed volume of 500 μL of the film, with concentrations of 0.158 mmol L^–1^, 0.316 mmol L^–1^, 0.474 mmol L^–1^ and 0.632 mmol L^–1^ of KMnO_4_, using phosphate buffer as a diluent to maintain a fixed total volume of 2 mL in the spectrofluorometer cuvette.

### 4.7 Thermodynamic Properties

To evaluate the effect of CQDs fluorescence intensity at different temperatures and identify the existing quenching mechanism, calibration curves were performed at 298.15, 300.15, and 303.15 K. In this procedure, fluorescence spectroscopy was used, keeping the volume of CQDs at 500 μL at their optimal concentration. Aliquots from 100 to 1000 μL of KMnO_4_ with a concentration of 2.53 mmol L^–1^ were added to a phosphate buffer solution to complete a total volume of 2 mL in the cuvette. For this, the Stern–Volmer model (eq 4) is used, from which the value of the K_SV_ constant could be obtained.

When more than one fluorescence quenching mechanism is identified, the Stern–Volmer equation is modified, according to eqs 5 and 6, highlighting the presence of the parameter *n*:^23^

4 $$\frac{I_{0}}{I}=1+K_{SV}\left[Q\right]$$

5 $$K_{SV}=K_{Q}\tau_{0}$$

6 $$\frac{I_{0}}{I}=1+K_{Q}\tau_{0}{[Q]}^{n}$$

where *I*_0_: fluorescence intensity in the absence of the quencher (Q); *I*: fluorescence intensity in the presence of the quencher (Q); *K*_*Q*_: bimolecular quenching constant; *τ*_*0*_: average fluorescence lifetime in the absence of the quencher; [Q]: quencher concentration; and *n* corresponds to the number of interaction sites or the number of molecules existing between the quencher and the fluorophore.

In the modified Stern–Volmer equation, the presence of parameter *n* is notable, which corresponds to the number of molecules or binding sites between the fluorophore and the quencher. To perform the thermodynamic analysis, the van’t Hoff equation (eq 7) is used. This equation is applied to analyze the relationship between the equilibrium constant (*K*_*Q*_) and temperature, allowing the identification of the type of interaction present, as well as obtaining the values of the system’s thermodynamic properties such as enthalpy (*ΔH°*), entropy (*ΔS°*), and Gibbs energy (*ΔG°*) calculated using eq 8:

7 $$\ln K_{Q}=-\frac{\Delta H^{{}^{\circ}}}{R}\frac{1}{T}+\frac{\Delta S^{{}^{\circ}}}{R}$$

8 $$\Delta G^{{}^{\circ}}=-RTlnK_{Q}$$

## Supplementary Material



## Notes

Figure S1, FTIR spectra of CQDs and precursors (citric acid and ethylenediamine); Figure S2. (a) TEM image and (b) size distribution of CQDs; Figure S3, fluorescence emission intensity at 450 nm for different excitation wavelengths of CQDs; Figure S4, fluorescence emission of CQDs as a function of concentration at 450 nm; Figure S5, absorbance spectrum of KMnO_4_, excitation spectrum of CQDs at 350 nm, emission spectrum of CQDs at 450 nm (PDF)
