# Supplementary material for: Fluorescent Nanocomposite Based on Carbon Quantum Dots for the Quantification of Permanganate Ions in Aquatic Environments
Source: ACS Omega. 2026 Jul 15;11(29):44263–74. doi: 10.1021/acsomega.6c04511 (PMC13425506; doi:10.1021/acsomega.6c04511)
Supplement: Supplementary file 1 [file ao6c04511_si_001.pdf]

Supplementary material for

Fluorescent nanocomposite based on Carbon Quantum Dots for the quantification of permanganate ions in aquatic environments

Ana Stephani Silva de Lima[1], Elayne Freitas de Carvalho[1], Carlos Mateus Paiva Oliveira[1], João Paulo de Sousa Ferreira[2], Renato Altobelli Antunes[3], Thiago Alves de Moura[4], Alexandre Rocha Paschoal[4], Juan Simón Rodriguez Hernández[4], Carlos William de Araújo Paschoal[4], Alejandro Pedro Ayala[4], Rafael Melo Freire[5], Pierre Basílio Almeida Fechine[1], Samuel Veloso Carneiro[1*]

[1]Advanced Materials Chemistry Group (GQMat), Department of Analytical Chemistry and Physical Chemistry, Federal University of Ceará – UFC, Campus do Pici, CP 12100, CEP 60451-970 Fortaleza, CE, Brazil

[2] Theoretical Chemistry Group (GQT), Department of Analytical Chemistry and Physical Chemistry, Federal University of Ceará – UFC, Campus do Pici, CP 12100, CEP 60451-970 Fortaleza, CE, Brazil

[3]Center for Engineering, Modeling and Applied Social Sciences, Federal University of ABC, CEP: 09210-580 - Santo André, SP – Brazil

[4]Department of Physics, Federal University of Ceará – UFC, Campus do Pici, CP 12100, CEP 60451-970 Fortaleza, CE, Brazil

[5]Laboratory of Nanomaterials Chemistry (LQN), Centro de Investigación en Ingeniería de Materiales (CIIMAT) y CEDENNA, Universidad Central de Chile, Santiago, CP 8330601, Chile

Ana Stephani Silva de Lima: anastephanilima01@gmail.com

Elayne Freitas de Carvalho: elaynefreitasc@alu.ufc.br

Carlos Mateus Paiva Oliveira: paivac805@gmail.com

João Paulo de Sousa Ferreira: joaopaulodesousaferreira16@gmail.com

Renato Altobelli Antunes: renato.antunes@ufabc.edu.br

Thiago Alves de Moura: thiagomoura@fisica.ufc.br

Alexandre Rocha Paschoal: paschoal@fisica.ufc.br

Juan Simón Rodriguez Hernández: juan.hernandez@fisica.ufc.br

Carlos William de Araújo Paschoal: paschoal.william@fisica.ufc.br

Alejandro Pedro Ayala: ayalla@fisica.ufc.br

Rafael Melo Freire: rafael.melo@ucentral.cl

Pierre Basílio Almeida Fechine: fechine@ufc.br

*Corresponding author: E-mail: samuel.veloso@ufc.br (Samuel V. Carneiro)

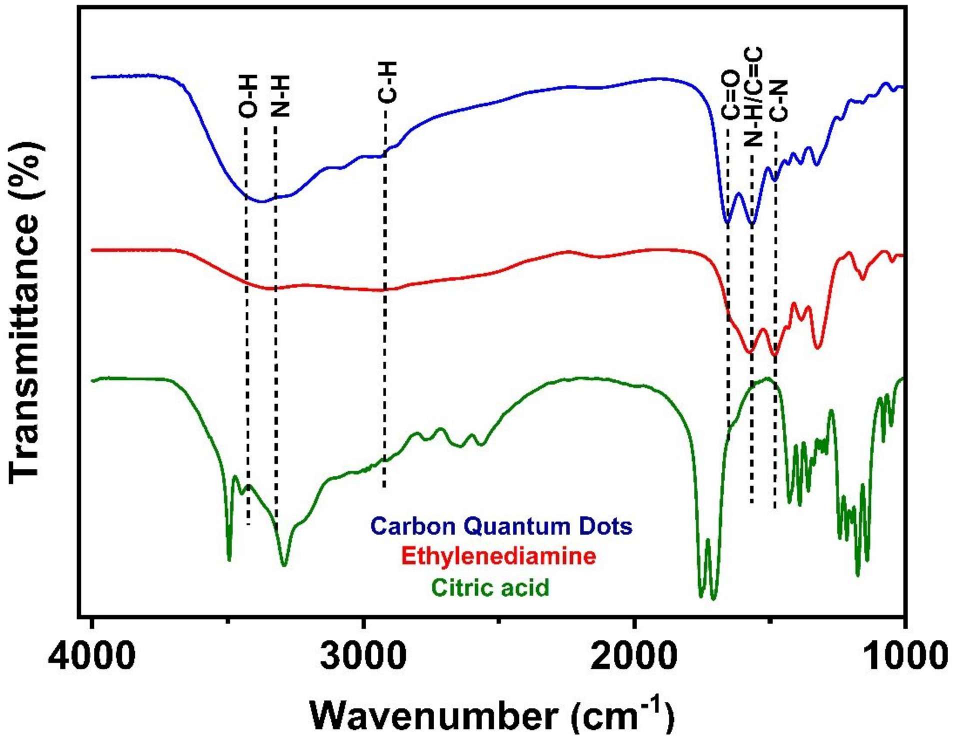

Fig. S1. FTIR spectrum of CQDs and the precursors citric acid and ethylenediamine.

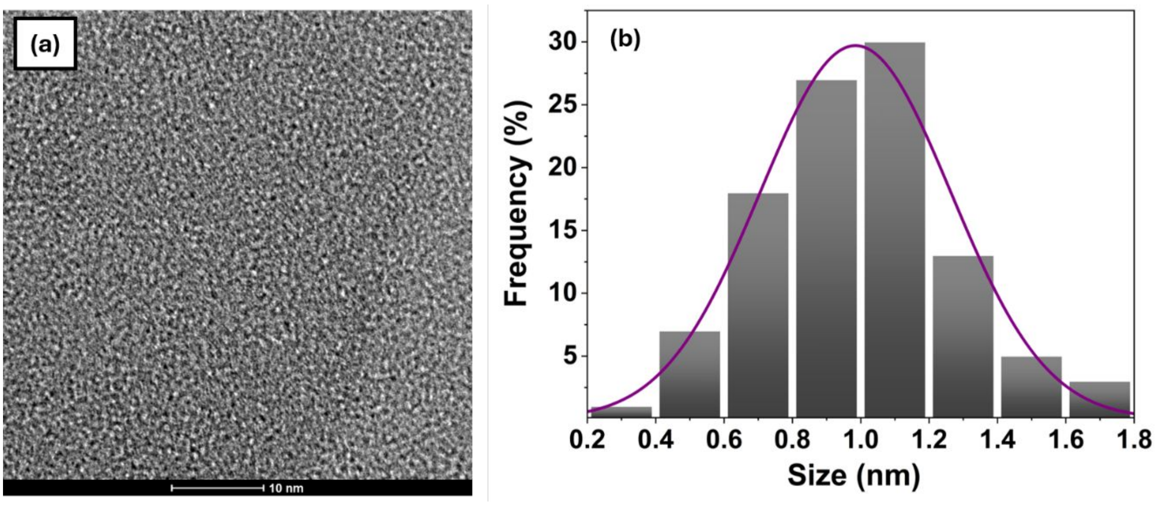

Fig. S2. (a) TEM image and **(b)** size distribution of CQDs.

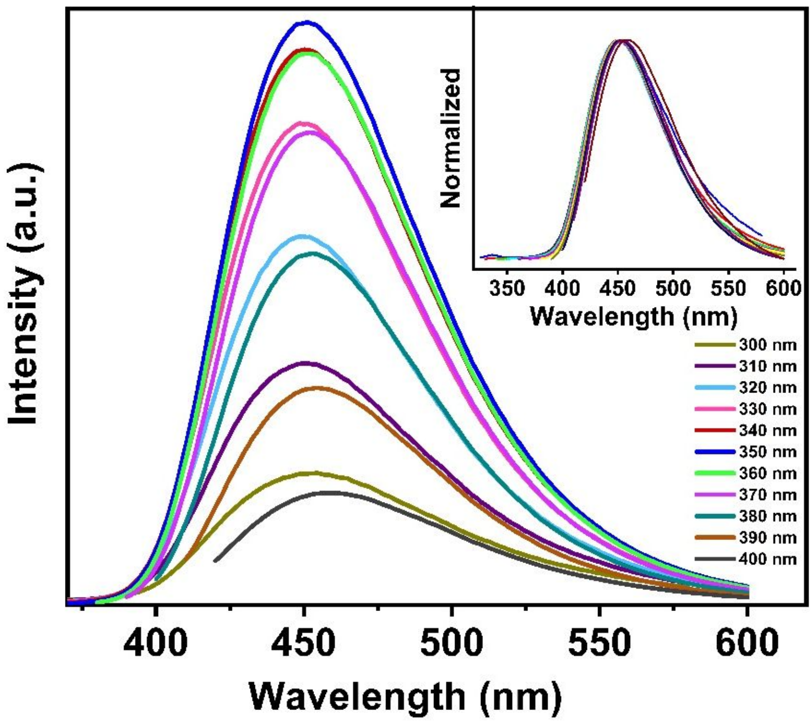

Fig. S3. Fluorescence emission intensity spectrum at 450 nm for different excitation wavelengths of the CQDs.

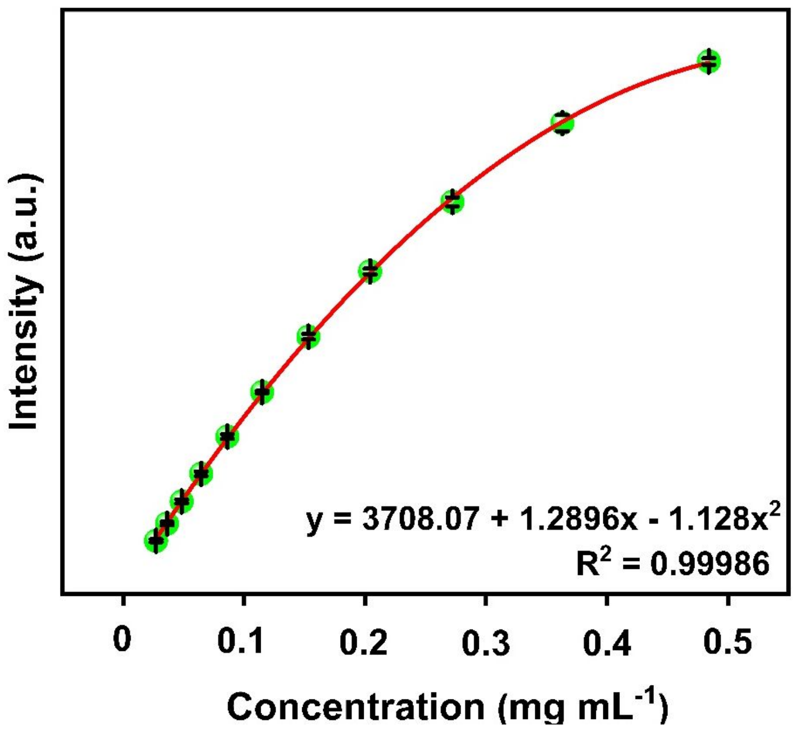

Fig. S4. Graph of CQDs fluorescence emission as a function of concentration at 450 nm.

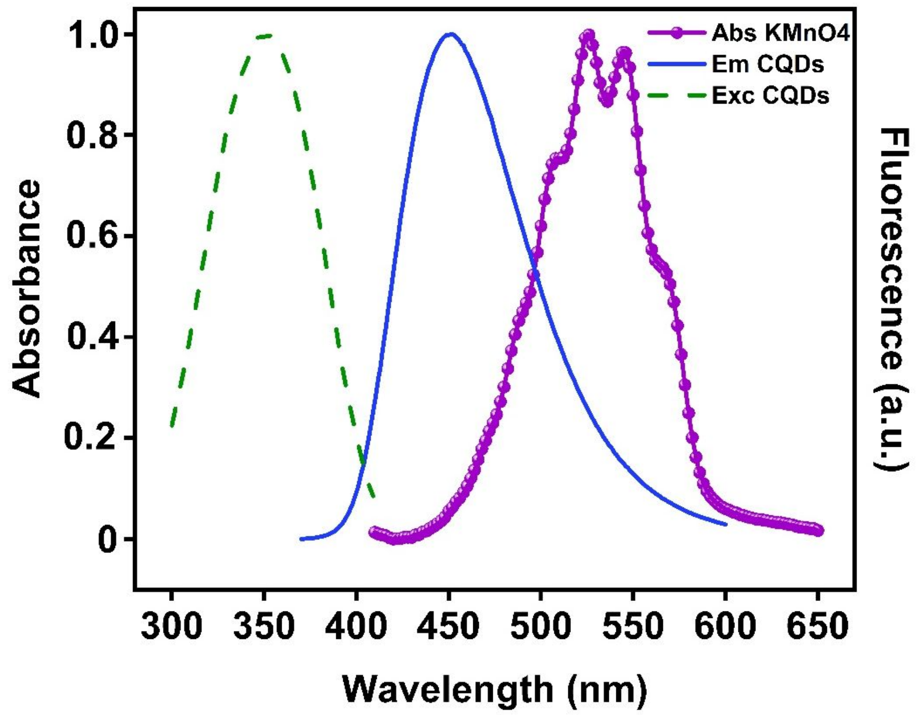

Fig. S5. Absorbance spectrum of $KMnO_4$, excitation spectrum at 350 nm of CQDs, emission spectrum at 450 nm of CQDs.
